# Association of levels of metabolites with the safe margin of rectal cancer surgery: a metabolomics study

**DOI:** 10.1186/s12885-022-10124-2

**Published:** 2022-10-05

**Authors:** Shaopeng Zhang, Guoqiang Pan, Zhifeng Liu, Yuan Kong, Daguang Wang

**Affiliations:** Department of Gastric and Colorectal Surgery, General Surgery Center, The First Hospital of Jilin University, 71 Xinmin Street, Changchun, Jilin, 130021 P.R. China

**Keywords:** Rectal cancer, Liquid chromatography–mass spectrometry, Safe margin, Metabolites

## Abstract

**Background:**

Rectal cancer is one of the most lethal of gastrointestinal malignancies. Metabonomics has gradually developed as a convenient, inexpensive and non-destructive technique for the study of cancers.

**Methods:**

A total of 150 tissue samples from 25 rectal cancer patients were analyzed by liquid chromatography–mass spectrometry (LC–MS), and 6 tissue samples were collected from each patient (group 1: tumor; group 2: 0.5 cm from tumor; group 3:1 cm from tumor; group 4:2 cm from tumor; group 5:3 cm from tumor and group 6:5 cm from tumor). The differential metabolites of tumor tissues and 5 cm from the tumor (normal tissues) were first selected. The differential metabolites between tumor tissues and normal tissues were regrouped by hierarchical clustering analysis, and further selected by discriminant analysis according to the regrouping of clustering results. The potential safe margin of clinical T(cT)1,cT2 stage rectal cancer and cT3,cT4 stage rectal cancer at the metabolomic level was further identified by observing the changes in the level of differential metabolites within the samples from group 1 to group 6.

**Results:**

We found 22 specific metabolites to distinguish tumor tissue and normal tissue. The most significant changes in metabolite levels were observed at 0.5 cm (cT1, cT2) and 2.0 cm (cT3, cT4) from the tumor, while the changes in the tissues afterwards showed a stable trend.

**Conclusions:**

There are differential metabolites between tumor tissues and normal tissues in rectal cancer. Based on our limited sample size, the safe distal incision margin for rectal cancer surgery in metabolites may be 0.5 cm in patients with cT1 and cT2 stage rectal cancer and 2.0 cm in patients with cT3 and cT4 stage rectal cancer.

## Introduction

Rectal cancer is the third most prevalent malignant gastrointestinal tumor, and is a serious threat to human health. There are more than 2.2 million new cases of rectal cancer worldwide every year, which has become an important topic in cancer research [1–3]. The 5-year survival rate for patients with early-stage rectal cancer is 90%, while the survival rate for patients with advanced rectal cancer is only 8–9% [4]. Currently, laparoscopic surgery is still the predominant treatment modality, and the safe distance to the rectal margin is associated with recurrence and metastasis of rectal cancer [5]. Lower rectal cancer located within 8 cm from the anus should undergo low anterior resection, which is a major problem, and related studies have shown that the anastomotic recurrence rate after low anterior rectal resection is as high as 4% to 10% [6]. The reasons for the persistently high recurrence rate of local margins after rectal cancer surgery are inaccurate safety margins and circumferential margins [7–9]. Unfortunately, there is no exact standard for the length of the safety cut margin [10]. Mass spectrometry techniques can detect the basic features and material basis of life activities [11–15], which are directly related to the final outcome of these life activities [4, 16]. There have been a large number of studies using mass spectrometry techniques in the evaluation of tumor incisions margins for breast, pancreatic, liver, kidney, and oral squamous cancers [17–20]. Our study used mass spectrometry chromatography analysis to identify the boundary line between rectal cancer tumor tissue and normal tissue, to provide a theoretical basis for safe incision margins in rectal cancer surgery.

## Materials and methods

### Patients

The present study was approved by the Ethics Committee of the First Hospital of Jilin University (Changchun, China), and all patients provided written informed consent. In this experiment, 150 rectal tissue samples were obtained from 25 rectal cancer patients (including: 2 patients with cT1 stage, 3 patients with cT2 stage, 16 patients with cT3 stage, and 4 patients with cT4 stage) who attended the Department of Gastrocolorectal and Anal Surgery, Baiquan First Hospital of Jilin University from July 2020 to November 2020. Inclusion criteria: (1) patients with solitary rectal cancer without distant metastases; (2) no previous oncologic history; (3) this was the first diagnosis; (4) no significant acute inflammatory disease. Exclusion criteria: (1) hepatic, renal, cardiopulmonary insufficiency; (2) pregnancy, female patients during lactation; (3) long-term alcohol and drug addiction; (4) long-term use of steroid hormones or nonsteroidal anti-inflammatory drugs; (4) chronic inflammatory diseases such as ulcerative colitis; (5) acute inflammatory reactions within the last 2 weeks; (6) major stress reactions such as major surgery, major changes or trauma within the last 2 weeks; (7) hematologic diseases such as leukemia; (8) infectious diseases.

### Sample collection

In each case, a fresh rectal cancer surgical sample was incised along the longitudinal axis of the intestinal canal and washed with saline to remove the intestinal contents and mucus on the surface of the intestinal mucosa. The washed sample was spread and laid flat on a plate, vascular forceps were clamped on the margin of the distal intestinal canal of the tumor, and the intestinal canal was torn flat with moderate force to facilitate resection. The length of the rectal intestinal canal was measured, and 0.5 cm diameter of whole intestinal wall tissue was cut at tumor tissue, 0.5 cm from tumor, 1 cm from tumor, 2.0 cm from tumor, 3 cm from tumor, and 5 cm from tumor. The samples were placed in an EP tube and immediately stored in a -80 °C refrigerator. The frozen tissue sample was allowed to thaw for 20 min at room temperature prior to analysis. The 10 mg of sample added 200 µl of water, and 800 µl of methanol:acetonitrile (1:1, V/V) was vortexed for 30 s, sonicated for 10 min (ice bath) and placed in a -20 °C refrigerator for 1 h. Subsequently, the samples were centrifuged at 13,500 × g/min for 15 min at 4 °C, and the supernatant was removed in a vacuum dryer until it evaporated. Then, 100 µl of acetonitrile:water (1:1, V/V) was added again, followed by vortexing for 30 s, sonicating for 5 min (ice bath), centrifuging at 13,500 × g/min for 15 min at 4 °C, and collecting the supernatants for LC–MS analysis. The quality control (QC) group consisted of all 150 samples, and each QC sample was inserted evenly into 10 experimental samples to assess the accuracy of the experiment. To ensure the accuracy and authenticity of the experimental data, the automatic calibration uses the chromatographic data system (CDS), which was used every fifth experimental sample.

## LC–MS

LC–MS analysis was performed using the AB Sciex TripleTOF 5600 system (Sciex) and Exion Ultra Performance Liquid Chromatography (UHPLC) system (Shimadzu, Japan) according to the manufacturer's protocols. The samples were mixed using a Vortex 3, Germany IKA; the centrifuge was a 4 °C low temperature centrifuge H165R from Xiangyi Centrifuge. Experimental reagents included acetonitrile (chromatographically pure, Sigma–Aldrich, America), formic acid (chromatographically pure, Sigma–Aldrich, America), methanol (chromatographically pure, Merk, Germany), deionized water (Watson, China), Positive Calibration Solution(AB Sciex) and Negative Calibration Solution(AB Sciex). Liquid chromatography was performed on an Exion UHPLC System. The column was an ACQUTIY UPLC HSS T3 (2.1 × 100 mm; pore size, 1.8 µm; Waters, America) maintained at 35 °C during separation. The mobile phases in positive ion mode were 0.1% formic acid in (A) water and (B) acetonitrile; the mobile phases in negative ion mode were (A) water and (B) acetonitrile. Samples were eluted with 98% A and 2% B for the first 0.5 min, a gradient from 2% B to 95% B over 10.5 min, followed by 95% B for 4 min. Then, the percentage of B was dropped to 2% within 0.1 min and maintained for 5 min. The flow rate was constant at 350 µl/min.

### Mass spectrometry

Mass spectrometry was performed using the AB Sciex TripleTOF 5600 system, which was fitted with an electrospray ionization source operating in positive and negative ion modes. Mass spectrometry conditions: the ion source level mass spectrometry parameters are as follows: Positive mode, IonSpray voltage (V): 5500; Temperature (℃): 550; Gas 1 (psi): 55; Gas 2 (psi): 55; Curtain gas (psi): 30; Declustering potential (DP): 100; Collision energy (CE): 10. Negative mode, IonSpray voltage (V): -4500; temperature (°C): 550; gas 1 (psi): 55; gas 2 (psi): 55; curtain gas (psi): 30; declustering potential (DP): -100; collision energy (CE): -10. The ion source secondary mass spectrometry parameters are as follows: Positive mode, IonSpray Voltage (V):5500;Temperature (℃):550;Gas 1 (psi):55;Gas 2 (psi):55; Curtain gas (psi): 30; Declustering Potential (DP):100; Collision Energy (CE):35;Collision Energy Spread (CES):15;Ion Release Delay (IRD):67;Ion Release Width (IRW):25;Negative mode, IonSpray Voltage (V):-4500;Temperature (℃):550;Gas 1 (psi):55;Gas 2 (psi):55; Curtain gas (psi): 30; Declustering Potential (DP):-100; Collision Energy (CE):-35;Collision Energy Spread (CES):15; Ion Release Delay (IRD): 67; Ion Release Width (IRW): 25.

### Statistical analysis

The LC–MS data were acquired using TF 1.7.1 software (AB Sciex LLC) and processed using PeakView software 2.2 (AB Sciex LLC). Normalization, scaling, noise filtering and peak alignment were performed using MarkerView software 1.3.1 (AB Sciex LLC). MetaboAnalyst 5.0 software was used to perform the principal component analysis (PCA), T test (*P* < 0.05 was considered to indicate a statistically significant difference), partial least squares discriminant analysis (PLS-DA), support vector machine discriminant analysis (SVMDA) and hierarchical clustering analysis to identify metabolites. SPSS 20.0 software (IBM Corp.) was used to generate receiver operating characteristic (ROC) curves. The mass-to-charge ratio (M/S) of the metabolites was imported into the Human Mass Spectrometry Database (HMDB) to find their material structure. The mean levels of the metabolites within 6 groups (1, 2, 3, 4, 5, 6) were plotted as a scatterplot to observe the trend of the levels of these metabolites with distance from the incision margin.

## Results

### Metabolomic profiles of tumor tissue and 5 cm from tumor (normal tissue)

**Fig. 1:**
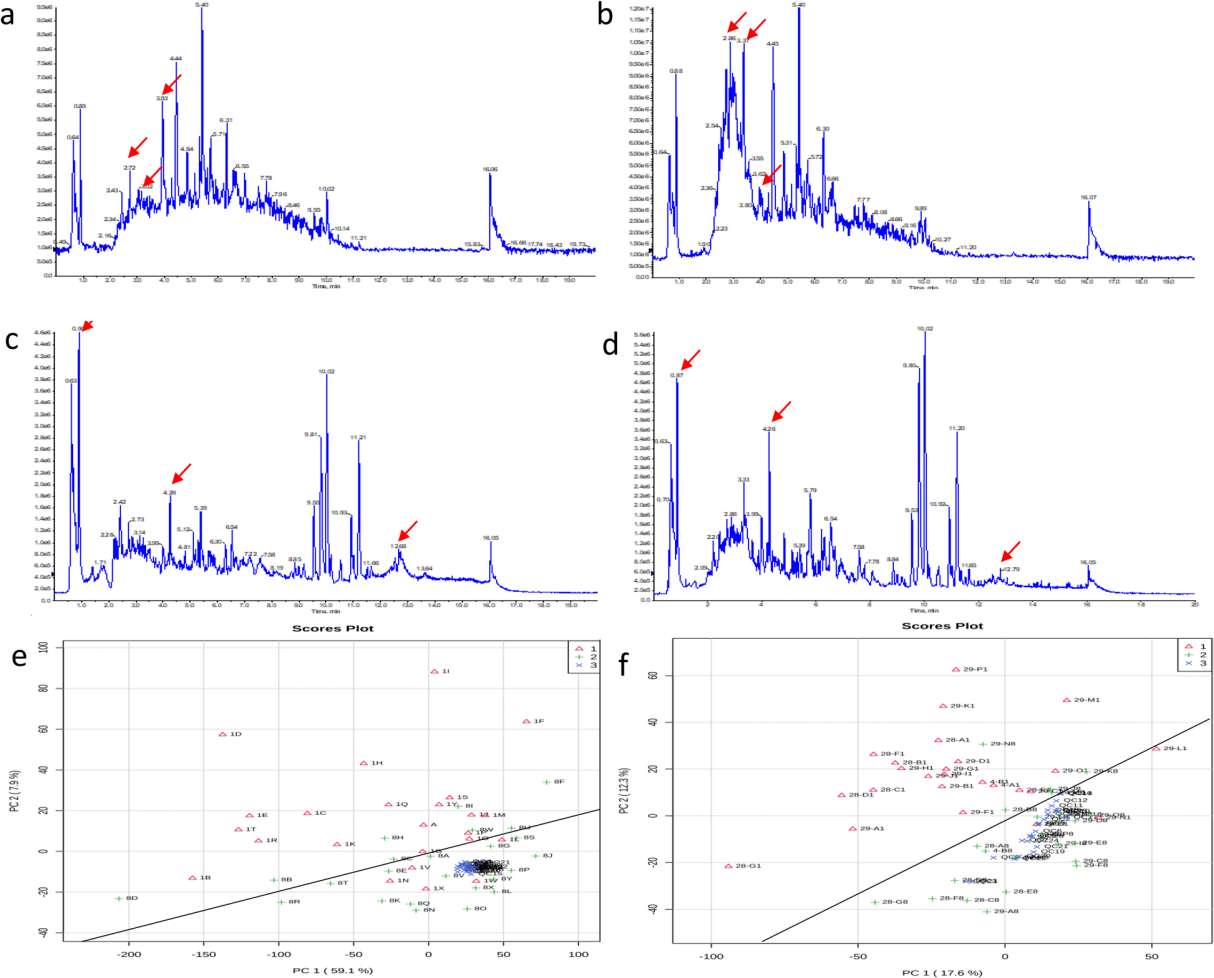
TIC chromatograms of metabolites and principal component analysis of samples of tumor tissue and 5 cm from tumor (normal tissue). **a** TIC profiles of tumor tissue in the positive ion mode; (**b**) TIC profiles of normal tissue in the positive ion mode. **c** TIC profiles of tumor tissue in the negative ion mode; (**d**) TIC profiles of normal tissue in the negative ion mode. Red arrows indicate metabolites subsequently identified as having different abundances in the two groups. **e** Principal component analysis in positive ion mode. **f** Principal component analysis in the negative ion mode. Each dot represents a single sample. Red represents the tumor tissue, green represents the normal tissue, and blue represents the QC group. TIC, total ion current. QC, quality control group

Representative total ion current chromatograms (TIC) of metabolites in the tumor tissue (Fig. 1a c) and 5 cm from the tumor (normal tissue) (Fig. 1b, d) were analysed. These results indicated differences in the levels of multiple metabolites in the positive ion mode (Fig. 1a, b) and negative ion mode (Fig. 1c, d). Additionally, the metabolomic profiles of tumor tissue and 5 cm from the tumor (normal tissue) were compared using PCA (Fig. 1e, f). The results demonstrated a clear separation of tumor tissue, 5 cm from tumor (normal tissue) and the QC sample in the positive ion mode (Fig. 1e) and negative ion mode (Fig. 1f). The difference in the type and number of metabolites determined the dispersion of the samples. The difference in metabolites in the samples was larger, with a greater distance between samples. In contrast, the smaller the difference in metabolites in samples, the closer the distance between samples. The QC sample consisted of 150 samples. The metabolites in the samples of the QC group were completely consistent, and the aggregation degree of these samples reflected the reliability of the experimental results. This result indicated that the distance between samples within each group was small, and therefore, the difference in metabolites in the group was also small. However, the distance of the samples between groups was large, indicating that the difference in metabolites between groups was large.

**Fig. 2:**
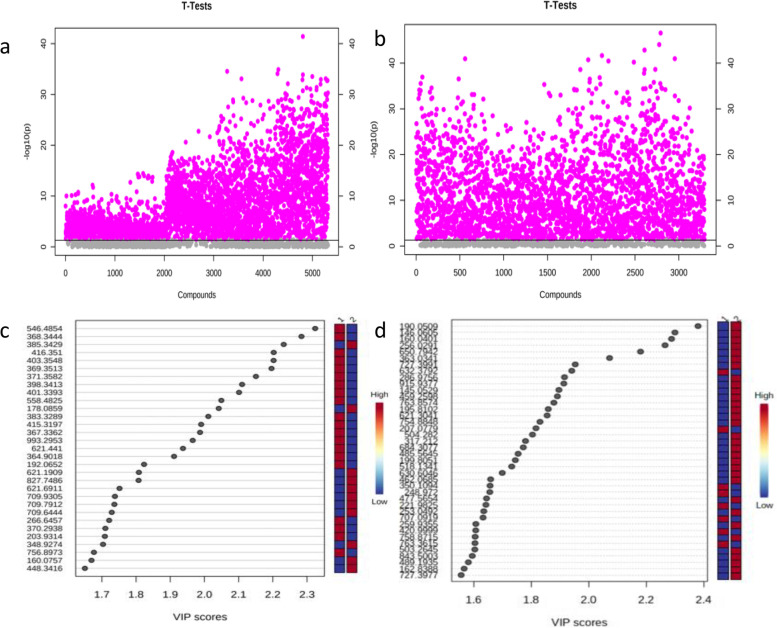
T test analysis and the VIP score plots of PLS-DA of samples of tumor tissue and 5 cm from tumor (normal tissue). **a** T test analysis in positive ion mode. **b** T test analysis in negative ion mode. The vertical coordinate is the *P* value, the horizontal coordinate is the level of each metabolite, and purple represents the significant metabolites with *P *< 0.05. **c** The VIP score plots of PLS-DA in positive ion mode. **d** The VIP score plots of PLS-DA in negative ion mode. The vertical coordinate represents the M/Z of each metabolite, and the horizontal coordinate is the weight value of each metabolite. It can be seen from the score plot that there are 31 metabolites in the positive ion mode and 40 in the negative ion mode. PLS-DA: partial least squares discriminant analysis. M/Z: mass-to-charge ratio. VIP: Variable Importance in the Projection

Comparison of the tumor tissue and 5 cm from the tumor (normal tissue) indicated significant differences for 458 metabolites in the positive ion mode (Fig. 2a) and 764 metabolites in the negative ion mode (Fig. 2b) (P < 0.05). Further screening was performed using PLS-DA to identify 31 differentiating metabolites with Variable Importance in the Projection(VIP) > 1 in the positive ion mode (Fig. 2c) and 40 differentiating metabolites with VIP > 1 in the negative ion mode (Fig. 2d).

Further screening was performed using SVMDA to identify 11 differentiating metabolites with weights of 100% in the positive ion mode and 11 differentiating metabolites with weights of 100% in the negative ion mode. Based on the HMDB (http://www.hmdb.ca), the following 22 metabolites met the criteria of the present study: Ethyl 3-Hydroxy Triester, Ditriglyceride, 7-Dehydrocholine, Acetamido phenylalamine, Triglyceride Monocelate, 24-O-B-D-Glycoside, Triglyceride, N-Ethanolamine, Propyl carnitine, Fatty Acetyl, Fatty Alcohol, 3,4-Dihydrooxyphenylpropanol, 4,5 Epoxy amide, 4-hydroxybutadibenzoic acid, 3'-phosphoamine sulfate 5'-phosphate, 1-O-β-D-glucosol-2,1-acetylglycerol phosphate, 1,2,3-triacetylglycerol,Amphetamine nucleoside monophosphate, adenosine acid, N-acetyl-α amino acid, and N-acrylamide (Table 1). Based on the measured levels of these 22 metabolites, 24/25 tumor tissues were classified as tumor tissues, and 23/25 normal tissues were classified as normal tissues (Table 2).

**Table 1:** Metabolites identified that differentiate tumor tissue and 5 cm from tumor tissue(normal tissue)

Ionization	m/z	Chemical formula	Metabolite
ESI +	558.4825	C15H30O3	Ethyl 3-Hydroxy Triester
ESI +	371.3582	C73H138O6	Ditriglyceride
ESI +	385.3429	C27H44O	7-Dehydrocholine
ESI +	756.8973	C70H127N3O29	Acetamido phenylalamine
ESI +	827.7486	C54H98O5	Triglyceride Monocelate
ESI +	621.1909	C36H60O8	24-O-B-D-Glycoside
ESI +	368.3444	C47H90O5	Triglyceride
ESI +	398.3413	C25H45NO	N-Ethanolamine
ESI +	993.2953	C32H56N7O18P3S	Propylcarnitine
ESI +	403.3548	C34H65NO3	Fatty Acetyl
ESI +	383.3289	C11H24O	Fatty Alcohol
ESI-	248.972	C8H10O4	3, 4-Dihydrooxyphenylpropanol
ESI-	199.8051	C40H58O4	4,5 Epoxy amide
ESI-	145.0529	C24H26N2O6	4-hydroxybutadibenzoic acid
ESI-	162.8388	ClH3O3	3' -phosphoamine sulfate 5' -phosphate
ESI-	195.8102	C35H58O7	1-O-β-D-glucosol-2
ESI-	504.282	C24H46NO9P	1-acetylglycerol phosphate
ESI-	253.0492	C9H14O6	1, 2, 3-triacetylglycerol
ESI-	707.0919	C9H13N2O9P	Amphetamine nucleoside monophosphate
ESI-	462.0685	C14H18N5O11P	adenosine acid
ESI-	684.3077	C26H37N9O6	N-acetyl-α amino acid
ESI-	650.7942	C59H89N19O13S	N-acrylamide

**Table 2:** Performance of 22 identified metabolites^a^ in differentiating tumor tissue and 5cm from tumor tissue(normal tissue)

Prediction	Reality		Total	Sensitivity	Specificity
	Tumor tissue	Normal tissue			
Tumor tissue	24	1	25		
Normal tissue	2	23	25	96.0%	92.0%
Total	26	24	50		

^a^The 22 metabolites included: Ethyl 3-Hydroxy Triester, Ditriglyceride, 7-Dehydrocholine, Acetamido phenylalamine, Triglyceride Monocelate, 24-O-B-D-Glycoside, Triglyceride, N-Ethanolamine, Propylcarnitine, Fatty Acetyl, Fatty Alcohol, 3, 4-Dihydrooxyphenylpropanol, 4,5 Epoxy amide, 4-hydroxybutadibenzoic acid, 3' -phosphoamine sulfate 5' -phosphate, 1-O-β-D-glucosol-2, 1-acetylglycerol phosphate, 1, 2, 3-triacetylglycerol, Amphetamine nucleoside monophosphate, adenosine acid, N-acetyl-α amino acid, N-acrylamide

**Fig. 3:**
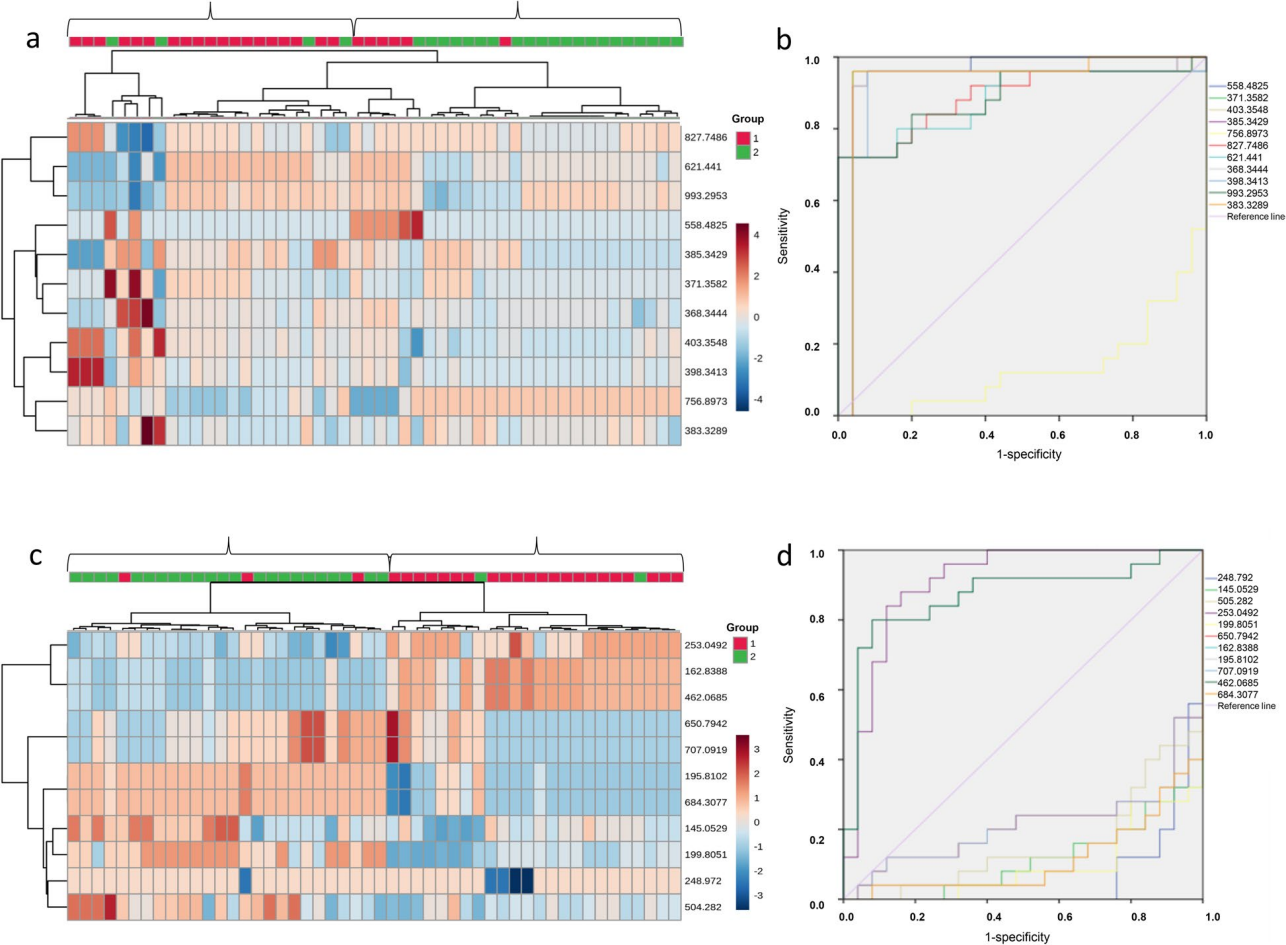
Hierarchical clustering analysis and ROC curves of metabolome data for metabolites in the 50 samples of tumor tissue and 5 cm from tumor (normal tissue). **a** Hierarchical clustering analysis in positive ion mode. **c** Hierarchical clustering analysis in negative ion mode. **b** ROC curves of the metabolites in the positive ion mode. **d** ROC curves of the metabolites in the negative ion mode. Metabolite levels were used to color code individual samples from blue (low concentration) to purple (high concentration). Tree clusters and shorter Euclidean distances indicate greater similarities between samples or metabolites. The red color of the top bars represents the tumor tissue, and the green color of the top bars represents the normal tissue. ROC, receiver operating characteristic

Therefore, this procedure had a sensitivity of 96.0% and a specificity of 92.0%. Hierarchical clustering analysis was also performed for these 22 metabolites to assess the similarity of the metabolomic profiles of the samples. The results indicated that the concentrations of these 22 metabolites separated the 50 samples into predominantly tumor tissue and 5 cm from tumor (normal tissue) subgroups in the positive ion mode (Fig. 3a) and in negative ion mode (Fig. 3c). ROC analysis was utilized to evaluate the performance of each metabolite as a marker for distinguishing tumor tissue from normal tissue (Fig. 3b, d).

### Analysis of differential metabolomic profiles in different tissues of patients with cT1, cT2 stage rectal cancer

**Fig. 4:**
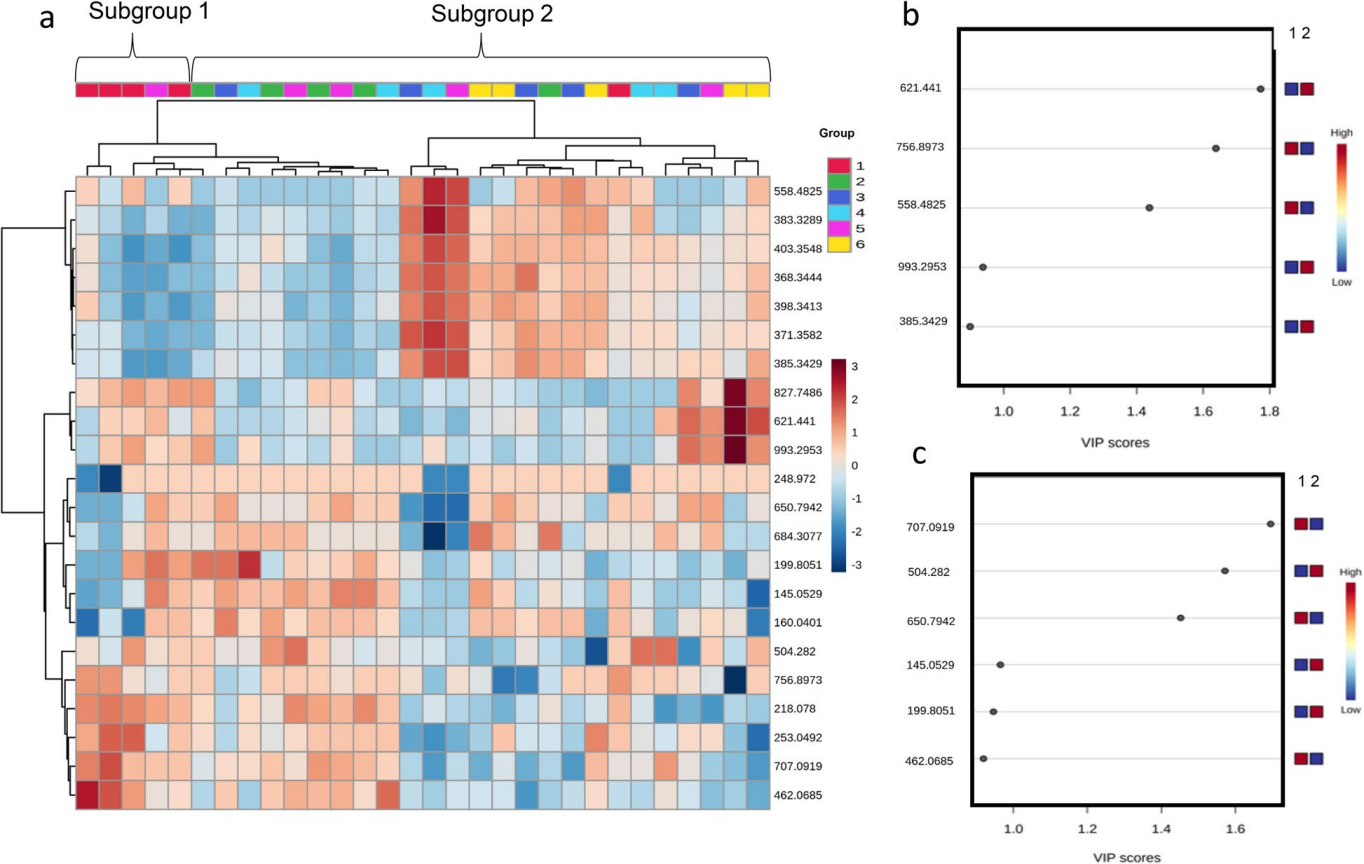
Hierarchical clustering analysis and VIP score plots of PLS-DA of 22 metabolites in 30 samples from 5 patients with cT1 and T2 stage tumors. **a** Hierarchical clustering analysis of 22 metabolites in 30 samples from 5 patients with cT1- and cT2-stage tumors. Metabolite levels were used to color code individual samples from blue (low concentration) to purple (high concentration). Tree clusters and shorter Euclidean distances indicate greater similarities between samples or metabolites. The red color of the top bars represents group 1, and the other color represents groups 2, 3, 4, 5, and 6. **b** The VIP score plots of PLS-DA in positive ion mode. **c** The VIP score plots of PLS-DA in negative ion mode. The vertical coordinate represents the M/Z of each metabolite, and the horizontal coordinate is the weight value of each metabolite. It can be seen from the score plot that there are 5 metabolites in the positive ion mode and 6 in the negative ion mode. PLS-DA: partial least squares discriminant analysis. M/Z: mass-to-charge ratio. VIP: Variable Importance in the Projection

Hierarchical clustering analysis was performed for these 22 metabolites in 30 samples from 5 patients with cT1 and cT2 stage tumors. This analysis separated these 30 samples from group 1–6 into subgroup 1 and subgroup 2 as shown in the top of Fig. 4a. The subgroup 1 included 4 samples from group 1 and 1 sample from group 4. The subgroup 2 included 24 samples from group 2, 3, 4, 5, and 6 and 1 sample from group 1 (Fig. 4a). According to the above subgroup, further screening was performed using PLS-DA to identify 5 differentiating metabolites with VIP > 0.8 in the positive ion mode (Fig. 4b) and 6 differentiating metabolites with VIP > 0.8 in the negative ion mode (Fig. 4c).

These 11 differentiating metabolites are shown in Table 3. Based on the measured levels of these 11 metabolites, 4/5 tumor tissue were classified as tumor tissue, and 21/25 ≥ 0.5 cm from tumor tissue were classified as ≥ 0.5 cm from tumor tissue (Table 4).

**Table 3:** Metabolites identified that differentiate tumor tissue and ≥ 0.5 cm from tumor tissue in cT1 and cT2 stage rectal cancer

Ionization	m/z	Chemical formula	Metabolite
ESI +	756.8973	C70H127N3O29	Acetamido phenylalamine
ESI +	558.4825	C15H30O3	Ethyl 3-Hydroxy Triester
ESI +	385.3429	C27H44O	7-Dehydrocholine
ESI +	993.2953	C32H56N7O18P3S	Propylcarnitine
ESI +	621.441	C36H60O8	24-O-B-D-Glycoside
ESI-	707.0919	C9H13N2O9P	Amphetamine nucleoside monophosphate
ESI-	504.282	C24H46NO9P	1-Acetylglycerin Phosphoric Acid
ESI-	650.7942	C59H89N19O13S	N-acrylamide
ESI-	145.0529	C24H26N2O6	4-hydroxybutadibenzoic acid
ESI-	199.8051	C40H58O4	4,5 Epoxy amide
ESI-	462.0685	C14H18N5O11P	adenosine acid

**Table 4:** Performance of 11 identified metabolites^a^ in differentiating tumor tissue and ≥ 0.5 cm from tumor tissue in cT1 and cT2 stage rectal cancer

Prediction	Reality		Total	Sensitivity	Specificity
	Tumor tissue	≥ 0.5 cm			
Tumor tissue	4	1	5		
≥ 0.5 cm	21	4	25	80.0%	84.0%
Total	25	5	30		

^a^The 11 metabolites included: Acetamido phenylalamine, Ethyl 3-Hydroxy Triester, 7-dehydrocholine, propylcarnitine, 24-O-B-D-Glycoside, Amphetamine nucleoside monophosphate, 1-Acetylglycerin Phosphoric Acid, N-Acrylamide, 4-hydroxybutadibenzoic acid, 4,5 Epoxy amide and adenosine acid

**Fig. 5:**
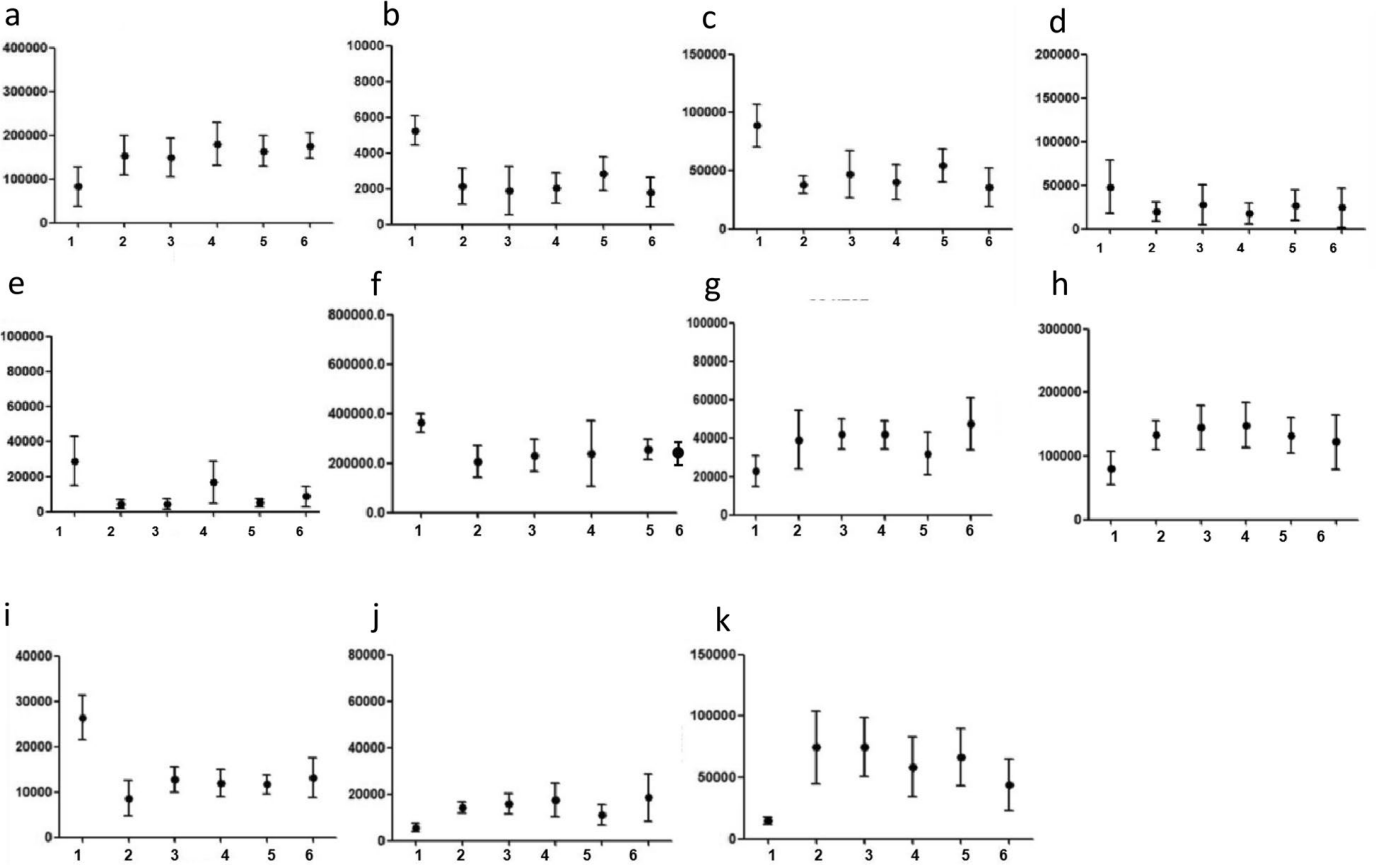
The mean levels of the 11 metabolites in 30 samples from 5 patients with cT1 and cT2 stage tumors. The most significant changes in metabolite levels were observed at 0.5 cm from the tumor, while the changes in the tissues afterwards showed a stable trend. a-k represent acetamido phenylalamine, ethyl 3-hydroxy Triester, 7-dehydrocholine, propylcarnitine, 24-O-B-D-glucoside, amphetamine nucleoside monophosphate, 1-acetylglycerin phosphoric acid, N-acrylamide, 4-hydroxybutadibenzoic acid, 4,5 epoxy amide and adenosine acid, respectively

Therefore, this procedure had a sensitivity of 80.0% and a specificity of 84.0%. Figure 5 shows the mean levels of the metabolites within 6 groups (1, 2, 3, 4, 5, 6) in patients with cT1 and cT2 rectal cancer. The most significant changes in metabolite levels were observed at 0.5 cm from the tumor, while the changes in the tissues afterwards showed a stable trend.

### Analysis of differential metabolomic profiles in different tissues of patients with cT3, cT4 stage rectal cancer

**Fig. 6:**
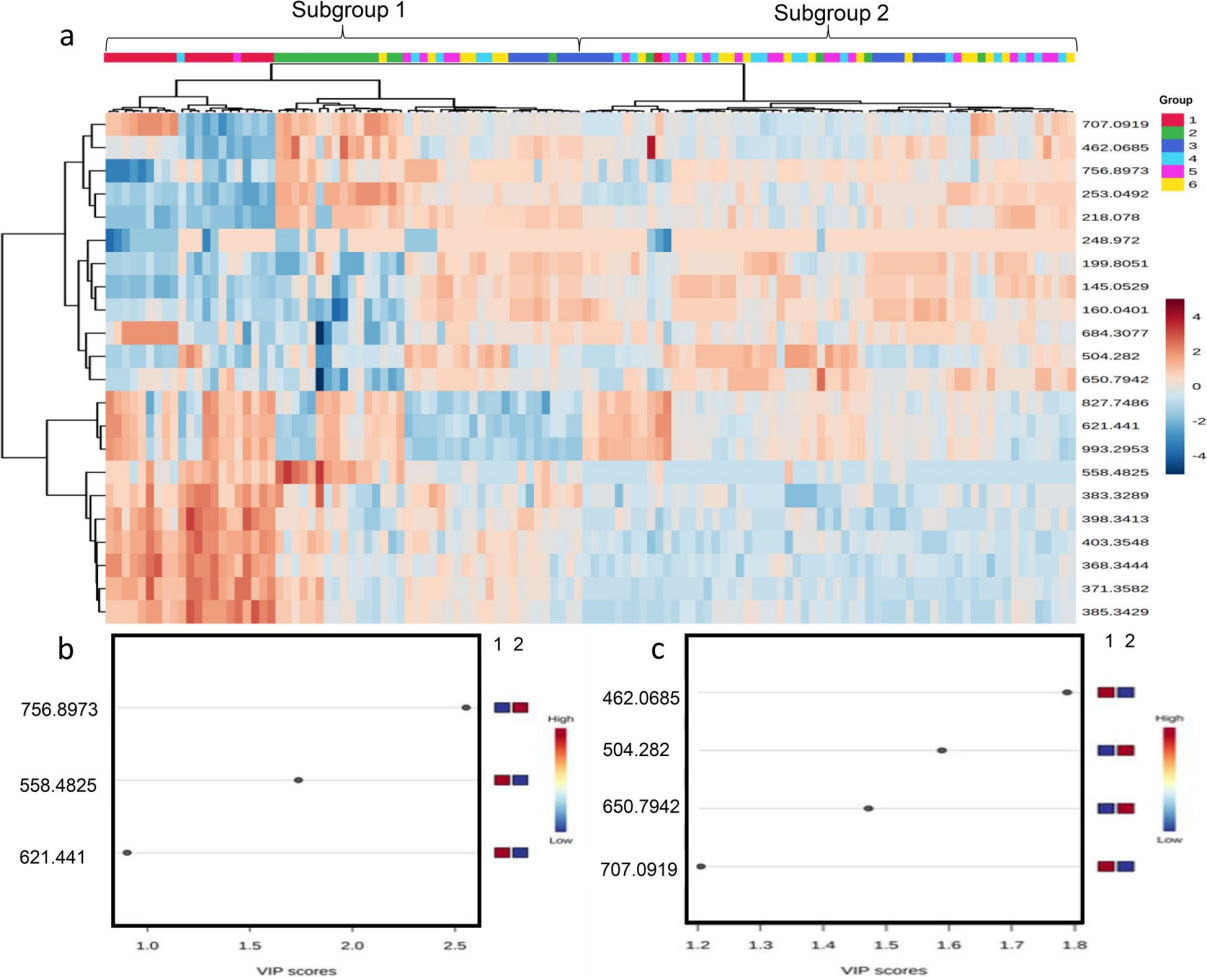
Hierarchical clustering analysis and VIP score plots of PLS-DA of 22 metabolites in 120 samples from 20 patients with cT3 and cT4 stage tumors. **a** Hierarchical clustering analysis of 22 metabolites in 120 samples from 20 patients with cT3- and cT4-stage tumors. Metabolite levels were used to color code individual samples from blue (low concentration) to purple (high concentration). Tree clusters and shorter Euclidean distances indicate greater similarities between samples or metabolites. The red, green, and blue colors of the top bars represent groups 1, 2, and 3, and the other colors represent groups 4, 5, and 6. **b** The VIP score plots of PLS-DA in positive ion mode. **c** The VIP score plots of PLS-DA in negative ion mode. The vertical coordinate represents the M/Z of each metabolite, and the horizontal coordinate is the weight value of each metabolite. It can be seen from the score plot that there are 3 metabolites in the positive ion mode and 4 in the negative ion mode. PLS-DA: partial least squares discriminant analysis

Hierarchical clustering analysis was performed for these 22 metabolites in 120 samples from 20 patients with cT3 and cT4 stage tumors. This analysis separated these 120 samples from group 1–6 into subgroup 1 and subgroup 2 as shown in the top of Fig. 6a. The subgroup 1 included 42 samples from groups 1, 2, and 3 and 16 samples from groups 4, 5, and 6. The subgroup 2 included 18 samples from group 1, 2, and 3 and 44 samples from group 4, 5, and 6 (Fig. 6a). According to the above subgroup, further screening was performed using PLS-DA to identify 3 differentiating metabolites with VIP > 0.8 in the positive ion mode (Fig. 6b) and 4 differentiating metabolites with VIP > 0.8 in the negative ion mode (Fig. 6c).

These 7 differentiating metabolites are shown in Table 5. Based on the measured levels of these 7 metabolites, 55/60 < 2 cm from tumor tissue were classified as < 2 cm from tumor tissue, and 51/60 ≥ 2 cm from tumor tissue were classified as ≥ 2 cm from tumor tissue (Table 6).

**Table 5:** Metabolites identified that differentiate < 2 cm from tumor tissue and ≥ 2.0 cm from tumor tissue in cT3 and cT4 stage rectal cancer

Ionization	m/z	Chemical formula	Metabolite
ESI +	756.8973	C70H127N3O29	Acetamido phenylalamine
ESI +	558.4825	C15H30O3	Ethyl 3-Hydroxy Triester
ESI +	621.441	C36H60O8	24-O-B-D-Glycoside
ESI-	462.0685	C14H18N5O11P	adenosine acid
ESI-	707.0919	C9H13N2O9P	Amphetamine nucleoside monophosphate
ESI-	504.282	C24H46NO9P	1-acetylglycerol phosphate
ESI-	650.7942	C59H89N19O13S	N-acrylamide

**Table 6:** Performance of 7 identified metabolites^a^ in differentiating < 2 cm from tumor tissue and ≥ 2.0 cm from tumor tissue in cT3 and cT4 stage rectal cancer

Prediction	Reality		Total	Sensitivity	Specificity
	< 2 cm	≥ 2 cm			
< 2 cm	55	5	60		
≥ 2 cm	9	51	60	91.6%	85.0%
Total	64	56	120		

^a^The 11 metabolites included: Acetamido phenylalamine, Ethyl 3-Hydroxy Triester, 24-O-B-D-Glycoside, adenosine acid, Amphetamine nucleoside monophosphate, 1-acetylglycerol phosphate and N-acrylamide

**Fig. 7:**
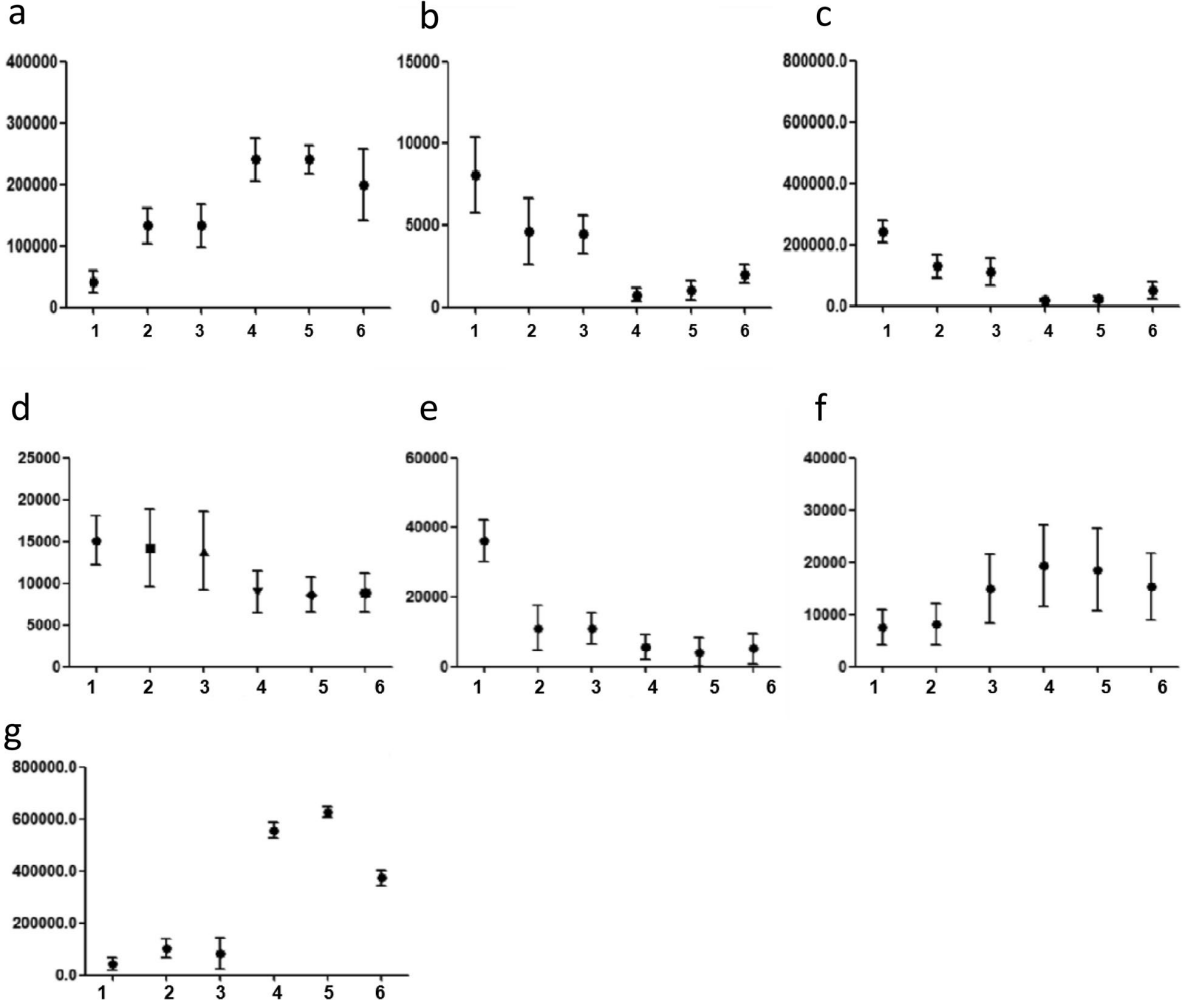
The mean levels of the 7 metabolites in 120 samples from 20 patients with cT3- and cT4-stage tumors. The most significant changes in metabolite levels were observed at 2.0 cm from the tumor, while the changes in the tissues afterwards showed a stable trend. a-g represent acetamido phenylalamine, ethyl 3-hydroxy Triester, 24-O-B-D-glycoside, adenosine acid, amphetamine nucleoside monophosphate, 1-acetylglycerol phosphate and N-acrylamide, respectively

Therefore, this procedure had a sensitivity of 91.6% and a specificity of 85.0%. Figure 7 shows the mean levels of the metabolites within 6 groups (1, 2, 3, 4, 5, 6) in patients with cT3 and cT4 stage rectal cancer. The most significant changes in metabolite levels were observed at 2.0 cm from the tumor, while the changes in the tissues afterwards showed a stable trend.

## Discussion

Rectal cancer is the third most common malignancy, and the distal margin distance is associated with local recurrence of the tumor, while there are conflicting opinions on the safe distal margin distance for low rectal cancer [5, 21–23].The National Comprehensive Cancer Network (NCCN) guidelines recommend a minimum of 5 cm of lower margins for patients with high rectal cancer and 1–2 cm of lower margins for patients with low rectal cancer [24]. The lower margin length of rectal cancer surgery has been reducing with the development of surgical procedures such as "closed incising" used in rectal cancer. Song found that a margin distance of less than 1 cm affected the local recurrence rate of progressive rectal cancer [25], while several other retrospective studies concluded that a distal margin of less than 1 cm was safe and did not increase the local recurrence rate or decrease the 5-year survival rate [26–28]. Zeng found that patients with rectal cancer with a distal rectal margin of less than 1 mm had a poorer prognosis [29]. We analysed the characteristics of metabolites in rectal cancer tissues and rectal tissues at 0.5 cm, 1 cm, 2 cm, 3 cm and 5 cm from rectal tumors by using LC–MS to observe the change levels of metabolites at different locations and further identify the possible safe incising margin. We found that the most significant changes in metabolite levels were observed at 0.5 cm from the tumor in patients with cT1 and cT2 stage rectal cancer, while the changes in the tissues afterwards showed a stable trend. The 0.5 cm may be the safe distal incision margin for rectal cancer surgery in metabolomics for patients with stage cT1 and cT2 stage rectal cancer. Some studies have shown that distal margins less than 1 cm are safe for patients with rectal cancer in terms of local recurrence rates and long-term surviva [26–28, 30, 31].We provide a theoretical basis for shorter distal margins, which are important for the preservation of anal function in low-grade rectal cancer. However,it should be acknowledged that the number of cT1 and cT2 samples in our study is limited with only 5 cases and our conclusion needs to be further confirmed by studies that contain larger sample sizes in the fulture.In patients with cT3 and cT4 stage rectal cancer, 2.0 cm may be the safe distal incision margin for rectal cancer surgery in metabolomics. We found that the safe distal margin distance increased with increasing tumor stage. Smith found that the invasion distance for ypT1 stage rectal cancer was 4 mm, while the invasion distance for ypT2 and ypT3 stage tumors was 9 mm by measuring the tumor invasion distance in rectal cancer receiving preoperative adjuvant radiotherapy [32]. Shimada reported the distal invasion distances of 4, 16, and 20 mm for stage I, II, and III rectal cancer [33].

Several previous metabolomic studies on rectal cancer have found changes in fatty acid metabolism, amino acid metabolism and glucose metabolism in patients with rectal cancer [34]. For example, Fernandes found significant differences in lipid profiles between rectal cancer patients and healthy individuals using mass spectrometry [35]. Wu identified differential metabolites in serum samples from patients with colon and rectal cancers by using gas chromatography–mass spectrometry(GC–MS) [36]. Our study identified 22 important differential metabolites that distinguish rectal cancer tissue and normal rectal mucosal tissue. Our study showed that the level of acetamido phenylalamine, a derivative of phenylalamine that acts as a precursor for the synthesis of epinephrine and tyrosine and regulates various physiological metabolisms of the body, was lower in the tumour tissue of rectal cancer patients than in normal tissue. A study found elevated serum phenylalanine levels associated with systemic inflammation, which plays a role in the pathogenesis of cancer cachexia by analysing the preoperative serum amino acid profile of 341 colorectal cancer patients [37]. It has also been found that serum phenylalanine levels are changed in patients with gastroesophageal cancer, possibly due to inflammation or other causes of phenylalanine hydroxylase dysfunction [38]. Carnitine is a quaternary ammonium compound biosynthesized from amino acids. Recent findings have suggested that carnitine system(CS) components are involved in the bidirectional transport of acyl moieties from cytosol to mitochondria and vice versa, thus playing a fundamental role in tuning the switch between glucose and fatty acid metabolism. Therefore, CS regulation, at both the enzymatic and epigenetic levels, plays a pivotal role in tumors [39]. A study found low carnitine levels in cancer cells from colorectal patients by analysing colorectal cancer cells and normal tissue cells, which may be related to low levels of organic cation/carnitine transporter 2 (OCTN2) in the cells [40]. The levels of propylcarnitine, which may be a derivative of carnitine, were lower in cancer tissue than in normal tissue in our study.

## Conclusions

There are differential metabolites, with levels varying with distance from the tumor, between tumor tissues and normal tissues in rectal cancer. In patients with cT1 and cT2 stage rectal cancer, the most significant changes in metabolite levels were observed at 0.5 cm from the tumor, while the changes in the tissues afterwards showed a stable trend. In patients with cT3 and cT4 stage rectal cancer, the most significant changes in metabolite levels were observed at 2.0 cm from the tumor, while the changes in the tissues afterwards showed a stable trend. Based on our limited sample size in our study, the safe distal incision margin for rectal cancer surgery in metabolites may be 0.5 cm in patients with stage cT1 and cT2 stage rectal cancer and 2.0 cm in patients with cT3 and cT4 stage rectal cancer. However, this conclusion needs to be further confirmed by studies that contain larger sample sizes in the fulture.

## Data Availability

The authors confirm that the data supporting the findings of this study are available within the article. Raw data that support the findings of the study are available from the corresponding author, upon reasonable request.
